# A review of urodynamic evaluation in children and its role in the management of boys with posterior urethral valves

**DOI:** 10.4103/0970-1591.36719

**Published:** 2007

**Authors:** Divyesh Y. Desai

**Affiliations:** Urodynamics Unit, Great Ormond Street Hospital for Children NHS Trust and University College London Hospitals, NHS Foundation Trust, Institute of Child Health, London, UK

**Keywords:** Children, posterior urethral valves, urodynamic evaluation

## Abstract

Posterior urethral valves are the commonest cause of lower urinary tract outflow obstruction in male infants with an estimated incidence of 1:5000 male infants and 1:25,000 live births. Despite treatment with fulguration of the obstructing valves, bladder function is abnormal in up to 70% of older children and adolescents. Bladder dysfunction causes morbidity e.g. urinary incontinence and has been implicated in the late deterioration of renal function in this population. A poor understanding and inappropriate management of bladder dysfunction can result in unnecessary morbidity, which can handicap a child for life. Any method that measures function or dysfunction of the lower urinary tract constitutes a urodynamics investigation. Broadly, the investigations can be classified into simple or noninvasive urodynamics and invasive urodynamics. The objective of urodynamics assessments in children is to reproduce the patient's complaints or symptoms. Video urodynamics can provide additional information that may contribute to a further understanding of the problem under investigation. Urodynamics provides a useful tool to test the efficacy of treatment as well as determine any refinements necessary to improve the outcome of such treatment.

Posterior urethral valves are the commonest cause of lower urinary tract outflow obstruction in male infants with an estimated incidence of 1:5000 male infants and 1:25,000 live births. Despite treatment with fulguration of the obstructing valves, bladder function is abnormal in up to 70% of older children and adolescents. A poor understanding and inappropriate management of bladder dysfunction can result in unnecessary morbidity, which can handicap a child for life.

Broadly, urodynamic investigations can be classified into two groups:

1. Simple or noninvasive urodynamics
2. Invasive urodynamics (cystometrography or CMG)

A full evaluation incorporating invasive urodynamics will define the pressure-volume relationships within the container in order to ascertain

1. At what pressures does the bladder store urine, i.e. an assessment of the compliance of the container.
2. The assessment would also try to ascertain whether or not there are any abnormal dynamics, over-activity or poor compliance that could be potentially detrimental to renal function and may be treated to reduce morbidity.
3. How does the bladder empty? The detrusor profiles and pressures during voiding, the relationship between detrusor contraction and sphincter function and the emptying efficiency of the container

Video urodynamics can provide additional information that may contribute to a further understanding of the problem under investigation.

Features that may become evident include:

1. Structural anomalies like diverticuli, obstructive lesions - residual valves, strictures etc.
2. Vesicoureteric reflux (VUR)- is it present during bladder filling, voiding or both? Is there any secondary refilling of the bladder?
3. Functional problems - detrusor-sphincter dis-coordination or suspected dysynergia
4. Bladder emptying, true and false residuals.

The more specialist applications of pediatric urodynamics are used to understand the evolving natural history of lower urinary tract function in specific pathological conditions:

1. Posterior urethral valves and
2. Vesicoureteric reflux

Urodynamics provide a useful tool to test the efficacy of treatment as well as determine any refinements necessary to improve the outcome of such treatment. Examples of this role of urodynamics can be found in

1. Evaluating the success of procedures to increase outlet resistance in patients with classic bladder exstrophy - Kelly repair
2. Assessing the effect of botulinum toxin injection in neuropathic detrusor overactivity
3. Assessing the impact of correction of VUR with bulking agents in early life on long-term outcome of bladder function.

Urodynamics have also been used in experimental studies in order to determine the effect of bladder outflow obstruction on the structure and function of the developing bladder. Finally, with an improved understanding of the causal factors in a urodynamic abnormality, urodynamic aids might be employed to direct treatment e.g. Biofeedback in the treatment of dysfunctional voiding disorders.

## NONINVASIVE URODYNAMICS

These assessments are performed in all children referred for evaluation. Following this initial assessment some children will go on to have an invasive urodynamics study.

### a. Frequency / Volume dairy

In children who are toilet trained, the diary is extremely useful in providing information regarding the nature of the problem in the child's home environment. This is compared with the clinical assessments that are performed in an environment, which is new to the child and can influence the results. The diary documents the periodicity and volumes of bladder emptying during the day, gives an idea about the functional bladder capacity in the daytime and the first void in the morning following a dry night usually reflects the maximum bladder capacity.

Furthermore, the diary can act as a positive feedback tool and a yardstick to assess efficacy of treatment (e.g. effectiveness of anticholinergic medication in the treatment of overactive bladder dysfunction).

### b. Interview

In addition to obtaining a good medical history the time spent with the child and family helps to evaluate the child's nature, his attitude to the problem being assessed, obtain information regarding his home and school environment and assess the child's compliance for proposed interventions.

### c. Bladder function assessment

This involves at least two free voids into a uroflow meter, observation of the child's posture during micturition noting whether there is any abdominal effort during voiding, post micturition dribbling and post void residual urine assessment using ultrasound. The flow pattern, rate, presence or absence of abdominal straining and voiding efficiency along with the frequency volume chart and history, gives useful information regarding bladder function or dysfunction. For further information regarding normal values and terminology please refer to the ICCS document on standardization of terminology.

This initial assessment must be carried out in all children referred for urodynamic evaluation. The information obtained will determine whether further invasive urodynamics are necessary and if so which type of study will best provide the missing information in order to direct management.

## INVASIVE URODYNAMICS

The various types of invasive studies include

- Natural filling urodynamics
- Standard artificial filling cystometrogram (CMG)
- Video contrast filling cystometrogram
- Isotope filling cystometrogram

The three commonly used studies are the natural fill; standard artificial fill and the video contrast fill studies.

### Techniques

In our unit, invasive urodynamic studies are performed via single or double lumen suprapubic catheters, which are placed under anesthesia at least 24h prior to the study. If the storage dynamics are the only critical aspect under evaluation the study may be carried out via a urethral catheter in children with impaired urethral sensation. Most centers within Europe perform invasive urodynamic studies via a urethral catheter and report similar findings; however, it must be recognized that assessment of the voiding dynamics in the presence of a urethral catheter, no matter how small its caliber will contribute additional resistance to outflow. In our experience when we have performed these studies via a urethral catheter we have found an error in estimating true outlet resistance and have usually overestimated it. This is of critical importance when one is planning reconstructive surgery for continence in children with neural tube defects. In addition voiding around a urethral catheter in children with normal urethral sensations may result in inhibition, affecting the interpretation of voiding bladder dynamics.

We also believe that it is far less traumatic to place the catheters under anesthetic and create the best possible environment in order to carry out the studies.

Abdominal pressure is usually recorded via a rectal catheter or alternatively a gastrostomy tube or a pressure recording catheter in the ACE channel if present can be used. Pelvic floor / sphincter activity can be recorded using surface or needle electrodes and the more recent urodynamic systems have demonstrated a more reliable ability to accurately record this activity.

Urodynamic evaluations in children are an important arm in the investigational algorithm of children with lower urinary tract dysfunction, and in order to obtain useful information from these studies it is very important to ensure the child's cooperation. Invasive urodynamics are difficult to perform and interpret, particularly in an uncooperative child and we believe that in order to obtain reliable and reproducible results, it is important to ensure

1. A fixed team, which comprises a Clinical Nurse Specialist, Urologist and Radiologist.
2. A rapport with the child by prior interaction with the child and family in order to familiarize the family with the environment and team.
3. Providing precise information regarding the test, what to expect and the information that is likely to be obtained.
4. Discuss possible problems or complications.
5. Explain the limitations of the type of test being used.

Interaction between staff, child and family prior to performing invasive tests can relieve anxiety, and a sense of familiarity, confidence and trust in what to expect goes a long way in ensuring maximal cooperation. A regular team of personnel minimizes the time involved in performing the test as each member has a clearly defined role and the experience gained over time will ensure optimal interpretation of test results.

These investigations are therefore best performed in an established unit that routinely carries out these tests in children in order to obtain results that are reliable, reproducible and help in determining optimum management.

## POSTERIOR URETHRAL VALVES

### Introduction

Antenatal ultrasounds detect urological anomalies in one per 800 live births; posterior urethral valves (PUV) account for 10% of these and antenatal diagnosis accounts for 50% of new cases of PUV suggesting a revised incidence of 1:4000 male births, a figure that will rise when nonviable and terminated fetuses are included.

Posterior urethral valve is a pan urinary tract disorder with a variable spectrum of severity affecting both the upper and lower urinary tract. Long-term follow-up studies have shown that the incidence of chronic and end stage renal failure were 34% and 10% respectively at 10 years of age and 51% and 38% at 20 years of age. End stage renal disease peaks twice during childhood, initially during the first year of life and this is generally attributed to inherent renal dysplasia. A second peak is seen in later childhood and adolescence, when the etiology is more complex and bladder dysfunction, seen in up to 70% of cases has been implicated in the pathophysiology of secondary renal damage and may account for late deterioration in renal function.

Antenatal diagnosis, together with successful management of early renal failure and respiratory distress and the efficacy of modern antibiotics have all contributed to lowering the early mortality from 50% in the 1950s to the present level of <3%. Focus has therefore now shifted towards tackling the long-term problems in this group. The long-term childhood morbidity in boys with treated PUV is largely due to

1. Late deterioration on renal function (50%) and
2. Bladder dysfunction (up to 70%)

### 1. Renal function

The causal relationship between dysplasia and PUV is unclear. It may be a primary event or may represent the final expression of fetal renal injury secondary to the abnormal urodynamics of obstruction.

Hennebery and Stephens[1] correlated histological findings of 22 renal units in boys with PUV with the position of the ipsilateral ureteral orifice. Severity of renal dysplasia increased with the more lateral ureteral orifice positions, leading them to conclude that their ‘bud theory’ described for primary VUR was applicable to PUV.

Alternatively, Bernstein[2] showed through experimental animal work that early unilateral ureteric obstruction in fetal lamb kidneys produced changes similar to the dysplastic changes seen in the kidneys of a human fetus with urinary tract obstruction. Whichever mechanism is responsible for dysplasia it is clearly an early embryological event that results in a fixed renal deficiency at birth.

### 2. Bladder dysfunction

The fetal bladder cyclically fills and empties from quite early on during development. During the last trimester the voiding frequency of the fetus is approximately 30 times in 24h. Normal bladder cycling generates stretch forces, which combine with a reduction in total collagen, ratio of collagen 3: Collagen 1 and a reduction in smooth muscle tension to produce a compliant container which stores adequate volumes of urine at low pressure. In PUV, normal bladder cycling fails.

Bladder dysfunction is thought to be responsible for delayed attainment and / or secondary onset of day or night time urinary incontinence and accounts for significant morbidity in a large proportion of boys with PUV. Whitaker *et al.*[3] found that only 29% of 112 boys were fully continent at a mean age of 6.5 years. Attainment of day and night continence is delayed in valve patients and was 19% at five years, 46% at 10 years and all except one at 20 years in Smith *et al*.'s[4] series. In the Parkhouse *et al*.[5] series 45% of five-year-olds were incontinent by day and 46% of these had a bad long-term outcome for renal function as defined by chronic renal failure (CRF) or end stage renal disease (ESRD).

The upper tract dilatation associated with urinary incontinence directed attention to the bladder and in 1980 Mitchell coined the term ‘valve bladder syndrome’ to describe a boy with a history of PUV, a noncompliant bladder, persistent upper tract dilatation and urinary incontinence. Peters[6] in 1990 published the results of urodynamic studies in 41 boys with PUV and urinary incontinence and described three main patterns.

1. Hypertonic low compliance, small capacity bladder.
2. Hyper-reflexic bladder with uninhibited contractions during filling that appeared to improve with time.
3. Myogenic failure with overflow and valsalva voiding.

## LONGITUDINAL STUDIES OF BLADDER FUNCTION IN BLADDER OUTFLOW IMPAIRMENT

### Experimental studies

Experimental studies have looked at the response of the bladder to outlet obstruction and Levin *et al*.s[7] elegant paper in 1995, describes the progression of the urinary bladder's response to experimentally induced partial outlet obstruction in rabbits to consist of three distinct phases.

### 1. Initial period (0-2 weeks)

There is a rapid progressive increase in bladder mass and a rapid increase in capacity. This is associated with a temporary loss in the bladder's ability to contract and thus generate pressure, which is rapidly restored followed by a slower progressive increase in its ability to sustain pressure and empty. Bladder function stabilizes by two weeks to enter the next stage, which has been described as the Compensated Phase.

### 2. Compensated phase

Bladder mass stabilizes, pressure generation increases above control levels but the *in vitro* ability of the bladder to empty remains reduced at between 70-80% of control levels. Also seen during this stage are progressive morphological changes in the distribution of both smooth muscle and collagen via the activated Angiotensin II and HB-EGF pathway which impair bladder capacity and compliance. Another feature noted in this phase is a partial de-nervation of the bladder, which is reversible on relief of obstruction. Following an ill-defined period of time bladder function destabilizes to enter the final stage.

### 3. Decompensated stage

During this stage there is a further progressive increase in bladder capacity, decrease in bladder compliance, contractility and its ability to empty. These abnormal lower tract dynamics over time can result in renal parenchymal compression, which can further damage inherently abnormal renal tissue.

### Clinical studies

**Figure 1:**
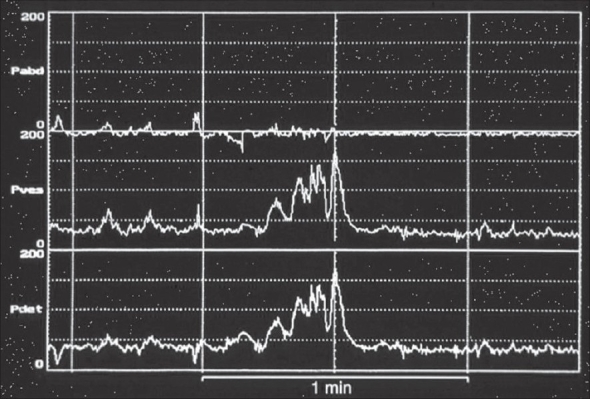
Natural Filling Urodynamics post valve resection in boys under two years of age Typical Features: a. Detrusor overactivity during bladder filling b. Reduced functional capacity c. High detrusor voiding pressures d. Incomplete bladder emptying

**Figure 2:**
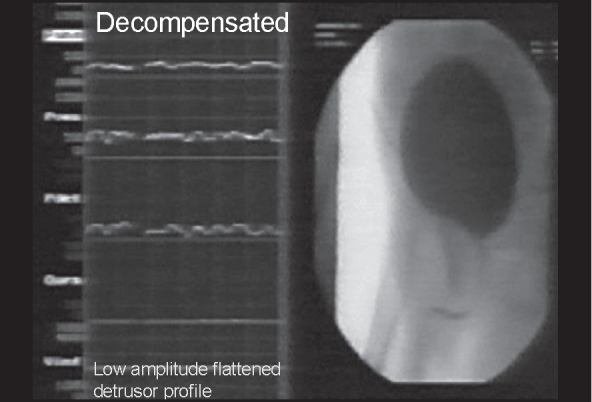
Decompensated Bladder Dynamics seen in teenage to early adult life. Typical features: a. Large capacity bladder which is stable b. Low detrusor voiding pressures c. Abdominal effort to initiate voiding d. Incomplete bladder emptying

Holmdahl *et al*.[8–10] have elegantly described an apparent evolution in urodynamics abnormalities with age in a series of papers (1995), which describe the changing urodynamic patterns of the valve bladder over time. Early on (0 - one year) the bladder has a reduced functional capacity, detrusor over-activity and is hyperreflexic [Figure 1]. Between one to three years of age bladder capacity increases, over-activity persists, hyper contractility is reduced and bladder emptying is incomplete. Later on between 4-12 years of age there is a further increase in capacity, absence of over-activity during filling, decrease in detrusor contractility during voiding with increased residual urine volumes. Holmdahl *et al*. also compared the results of urodynamic studies in six boys, pre and post puberty and found evidence of bladder de-compensation in all six, post puberty [Figure 2]. De Gennaro *et al*., in 2000[11] have subsequently described similar findings.

The identification of specific types of bladder dysfunction on urodynamic assessment provides an opportunity for treatment and improving symptoms. In addition it has the potential to protect renal function from secondary damage due to the abnormal bladder dynamics.

The Role of Urodynamics in the Management of Posterior Urethral Valves

The bladder in posterior urethral valves has been and continues to remain an enigma. Antenatal fetal shunting has gained momentum although the benefits of this approach are as yet unclear. In order to try and address this issue, at the present time there is an ongoing multicenter trial (PLUTO - UK and European centers), which will look at the outcome of vesico-amniotic shunting in a randomized controlled manner.

Churchill *et al*.[12] and Jauregiezar *et al*.,[13] have independently showed the benefits of proximal urinary diversion compared to valve ablation alone on the early outcome of renal function; however, the long-term outcome of renal function, following primary valve ablation or proximal urinary diversion is comparable. Extrapolating from this data and our knowledge of urodynamic bladder function in infancy following valve ablation, it would appear logical and reasonable to offer urinary diversion in selected cases with severely impaired renal function. This approach may delay the need for renal replacement to an age when it is easier to manage but will not change the long-term outcome of renal function in this group.

Clearly, boys with PUV need to be carefully monitored with regards to renal and bladder function status during early childhood, as toilet training and the attainment of urinary continence could possibly be delayed in this group. A proportion of boys with PUV will have normal bladder function but as yet there is no way in which this can be predicted in early life.

Indications for Invasive Urodynamic Studies in PUV

1. Persistent daytime urinary incontinence beyond the age of five years.
2. Deterioration in renal function (rising creatinine or drop in GFR) with no obvious cause like growth spurt etc.
3. Increase in upper tract dilatation in the absence of ongoing outflow obstruction.
4. Prior to Renal Transplantation - to ensure a safe, compliant low-pressure urinary tract.
5. To assess the efficacy of treatment or intervention.
6. As a research tool to study the evolution or natural history of developing bladder function in boys with successfully treated PUV.

## TYPES OF INVASIVE URODYNAMIC STUDIES

### 1. Natural filling urodynamics [Figure 3]

**Figure 3:**
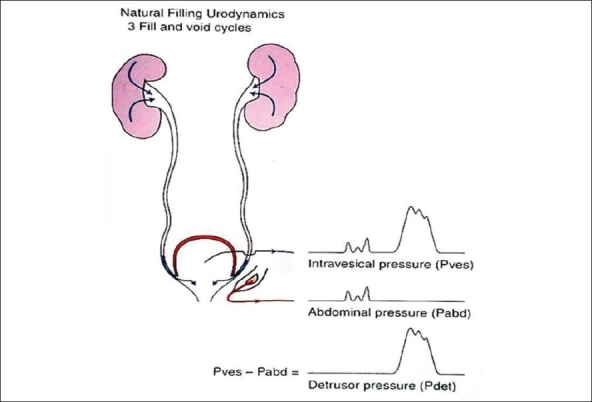
Natural filling urodynamics

This method is suitable to assess bladder function in infancy or early childhood and in situations where the bladder symptoms cannot be detected by conventional CMG. The bladder is allowed to fill naturally with urine produced by the kidneys. A single lumen catheter is used to record vesical pressures and a rectal catheter for abdominal pressure measurement. In general three filling and voiding cycles are required to establish reproducibility. This method of recording bladder activity is also described as a Physiological Fill Urodynamic Study.

#### Advantages

- Slow rate of bladder filling by urine, which is at body temperature minimizes the risk of inducing iatrogenic detrusor overactivity.
- The study can be carried out in ambulatory as well as overnight study settings.
- A small caliber catheter is sufficient to accurately record pressure changes thus minimizing the risk of bladder spasms
- Physiological

#### Disadvantages

- The study can take a long time in children with PUV with a large bladder capacity and storage of urine in the upper tracts.
- Synchronous fluoroscopic imaging not possible thus information regarding VUR, residual obstruction cannot be ascertained

### 2. Artificial fill urodynamics (Ward or Video)

The bladder is filled artificially with saline or radio contrast medium. A double-lumen or two single-lumen catheters are required, one for bladder filling and the other to record pressures. A rectal catheter is used to record abdominal pressures. The bladder is usually filled with the aid of gravity at a rate not exceeding 10-15ml/min. (Refer to ICCS document on terminology for further information.)

#### Advantages

- Time required to complete one cycle of bladder filling and emptying is short, even in cases where the bladder capacity is overtly large
- Synchronous fluoroscopic imaging possible giving additional information regarding VUR, diverticula, urethral anatomy, voiding efficiency and secondary refilling

#### Disadvantages

**Figure 4:**
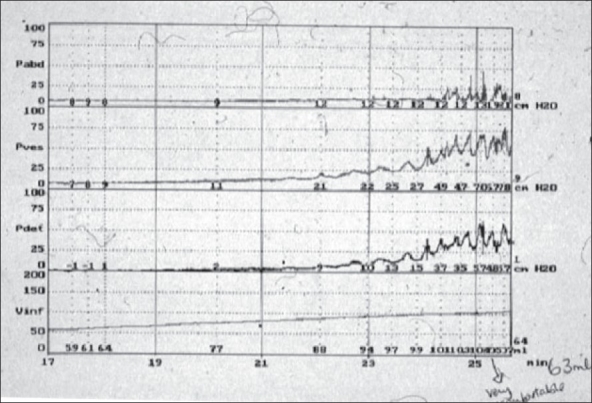
Artificial poorly compliant bladder with overactivity Can result from the following: a. Rapid rate of bladder filling b. Lowered temperature of filling solution c. Bladder overfilling beyond normal capacity
- Presence of a larger or two catheters may induce bladder spasms
- Requires a cooperative child who is able to communicate and respond to instructions.

- Temperature of the fluid used for bladder filling can influence the filling characteristics and fluids at less than body temperature can induce detrusor overactivity.
- Similarly a high rate of filling can result in detrusor overactivity and reduced bladder compliance [Figure 4]

Video urodynamics are the most commonly performed invasive studies in boys with PUV.

## PITFALLS

### 1. Vesicoureteric reflux

In cases where significant VUR is suspected, a video urodynamic study is the study of choice. A slow rate of filling with frequent intermittent fluoroscopic screening will help determine the severity of the problem. Vesicoureteric reflux may occur from the early stages of bladder filling (low-pressure reflux), in which case the pressures recorded will reflect system (bladder + upper tract) compliance and capacity. Vesicoureteric reflux may however appear towards the end of bladder filling or only during voiding (high-pressure reflux) and in these cases the VUR has no effect on the bladder's storage characteristics.

Similarly, incomplete bladder emptying may be the result of a failing detrusor or the result of secondary refilling from the upper tracts due to VUR and more rarely, a poorly compliant container.

### 2. After contraction

This urodynamic feature is frequently observed during invasive studies in children with PUV. It usually occurs as a sharp spike or a series of spikes at the end of micturition and should not be included in the analysis of the voiding phase of the study. The significance of this phenomenon has been debated and some believe it is representative of obstruction. An alternative explanation is that it represents the response of an empty bladder to the presence of catheters – a bladder spasm, further supported by the observation that after contractions are rarely seen in decompensated bladders.

### 3. Anticholinergics

It is not uncommon for boys with PUV to be treated with oxybutinin for urinary incontinence. Prior to performing the urodynamic study it is advisable to discontinue the medication 48h prior in order to establish the true nature of bladder function, i.e. presence or absence of detrusor overactivity.

### 4. Urethral instrumentation

Cystoscopy and any other instrumentation just prior to the study can influence both the filling and the voiding phase of the study and therefore if necessary must be carried out prior to the study on a separate occasion.

Similarly, it is prudent to wait for at least six to 12 months following valve resection prior to assessing bladder dysfunction. The compensatory changes in the bladder take time to resolve following relief of obstruction and early results of bladder function are likely to change with the passage of time.

## TREATMENT OF BLADDER DYSFUNCTION

### Overview

Strategies include pharmacotherapy, bladder drainage and surgical intervention. The assessment should include information regarding 24-h urine production and particularly the overnight urine volumes.

Clean intermittent catheterization (CIC) is an important adjunct in the treatment although in the presence of normal urethral sensation it may not be tolerated per urethra.

### Pharmacotherapy

Oxybutinin and more recently Tolterodine, have been the mainstay in the pharmacological management of valve bladders. The use of anticholinergics has been shown to be effective in treating overactivity during bladder filling and loss of bladder compliance.

Glassberg *et al*.[14] reported their experience with anticholinergic therapy in the treatment of 13 boys with incontinence due to poor compliance. Ten out of 13 became dry with dramatic improvements in the other three and 10/12 refluxing units stopped refluxing with anticholinergic medication.

One side-effect of anticholinergic therapy is incomplete bladder emptying and in severe cases myogenic failure and Glassberg found this to be reversible on stopping therapy.

Historically, secondary bladder neck obstruction in valve patients has been over diagnosed and bladder neck incisions have been associated with poor outcome. McGuire and Weiss were the first to address the role of non-selective alpha-blocker phenoxybenzamine to treat secondary bladder neck obstruction; however their use is at present limited.

Both Kim *et al*.[15] and Glassberg[16] have attributed symptoms of outflow impairment in a group of boys with treated PUV to bladder neck obstruction using urodynamic criteria. They have gone on to incise the bladder neck and have shown clinical and urodynamic improvement.

### Bladder drainage

#### Refluxing ureterostomy

The valve bladder in the first one to two years following valve ablation has a reduced capacity with detrusor overactivity and high voiding pressures. In boys with gross reflux and impaired renal function, a low refluxing ureterostomy, proposed by Philip Ransley is an elegant way of neutralizing the abnormal bladder dynamics and at the same time maintaining bladder cycling. The ureterostomy can be closed at a later date, alternatively if associated with a poorly functioning kidney proceed to a nephrectomy.

### CIC

Koff *et al*.,[17] reported on the efficacy of overnight bladder drainage in treating the over-distended bladder with persistent upper urinary tract dilatation. Overnight drainage allows the bladder to empty for part of the 24-h day, an opportunity these bladders do not otherwise have and relieves the pressure on the upper urinary tract for 8-10h. In addition it is a very effective way of dealing with bladder over-distension secondary to polyuria, which is frequently present in children with PUV. This may be difficult to achieve via urethral catheterization and consideration may be given to the Mitrofanoff procedure.

### Augmentation cystoplasty

In bladders with abnormal dynamics that are resistant to pharmacological manipulation and intermittent catheterization with evidence of detrimental effects on upper tract function, bladder augmentation with the placement of a Mitrofanoff conduit has been shown to be effective in selected cases.

A review of 20 patients[18] who underwent augmentation for reduced capacity and documented poorly compliant overactive bladders resistant to anticholinergic therapy, showed the potential of this approach in carefully selected cases. All 20 patients had a high 24-h urine output and an abnormal GFR. Augmentation was with ileum (nine), stomach (seven), colon (two) and ureter (two) and six patients had simultaneous placement of a Mitrofanoff conduit. Upper tract dilatation improved in 17 and stabilized in three cases. Seventeen patients became dry by day and night; three require overnight drainage for severe polyuria. Eleven voided spontaneously to completion; seven voided but catheterized to achieve satisfactory emptying and a further two remained dependent on catheterization only to achieve bladder emptying.

### Other interventions

In boys who progress to ESRD with polyuria, the high 24-h urine output can impact on hitherto stable bladder function and result in secondary incontinence. This change in bladder function can be reverted by native kidney nephrectomy with a subsequent reduction in 24-h urine output has shown to improve bladder emptying in carefully selected cases. A similar improvement in polyuria has been noted following successful renal transplantation.
